# Artificial Intelligence and Digital Microscopy Applications in Diagnostic Hematopathology

**DOI:** 10.3390/cancers12040797

**Published:** 2020-03-26

**Authors:** Hanadi El Achi, Joseph D. Khoury

**Affiliations:** 1 Department of Pathology and Laboratory Medicine, The University of Texas Health Science Center at Houston, Houston, TX 77030, USA; hanadi.s.elachi@uth.tmc.edu; 2 Department of Hematopathology, The University of Texas MD Anderson Cancer Center, Houston, TX 77030, USA

**Keywords:** digital pathology, artificial intelligence, leukemia, lymphoma, hematopathology

## Abstract

Digital Pathology is the process of converting histology glass slides to digital images using sophisticated computerized technology to facilitate acquisition, evaluation, storage, and portability of histologic information. By its nature, digitization of analog histology data renders it amenable to analysis using deep learning/artificial intelligence (DL/AI) techniques. The application of DL/AI to digital pathology data holds promise, even if the scope of use cases and regulatory framework for deploying such applications in the clinical environment remains in the early stages. Recent studies using whole-slide images and DL/AI to detect histologic abnormalities in general and cancer in particular have shown encouraging results. In this review, we focus on these emerging technologies intended for use in diagnostic hematology and the evaluation of lymphoproliferative diseases.

## 1. Introduction

Digital pathology (DP) is the process of converting histology glass slides to digital images enabled by sophisticated computerized technology to facilitate management and interpretation of histologic information using machine learning (ML) techniques [1]. Machine learning, a branch of artificial intelligence (AI), consists of algorithms and statistical models that automate the performance of particular tasks. The system learns from previously saved data to make predictions on new data with minimal human intervention and without using direct instructions.

**Figure 1:**
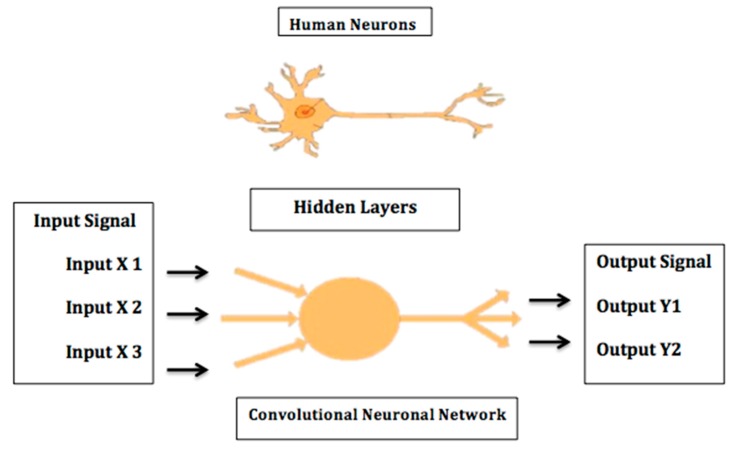
A neural network is a computer system modeled on the human brain.

ML frameworks have evolved considerably over the past few decades. Traditional methods include algorithms such as support vector machine (SVM), neural network (NN), logistic regression, random forest, and naïve Bayes. On the other hand, modern deep learning (DL) methods include convolutional neural networks (CNN or ConvNet), recursive neural networks, long short-term memory (LSTM), deep belief networks, convolutional deep belief networks, Boltzmann machines, stacked auto-encoders, tensor deep stacking networks, spike-and-slab RBMs, compound hierarchical-deep models, deep coding networks, deep q-networks, encoder–decoder networks, and multilayer kernel machine. DL algorithms are based on the *artificial neural network* principle, which emulates the structure and the function of human brain neurons (Figure 1). The neural network consists of thousands of layers of neurons, with each group of neurons that correspond to a certain layer receiving input from neurons of the underlying layer. Neurons subsequently use supervised, unsupervised, or semi-supervised learning to detect certain characteristic and pathognomonic features that are then stacked together and fine-tuned to generate a new output transmitted to the next layer [2,3].

Deep learning applications encompass a broad scope of disciplines, including image recognition, visual art processing, natural language processing, and bioinformatics. DL applications are gradually making their way into the healthcare system, particularly in medical devices and electronic record networks. For instance, ML is integrated into state-of-the-art robotic-assisted surgery. Automated knot-tying using recurrent neural networks (RNN) was employed by Mayer et al.; this method showed impressive results in speeding up knot-tying and ultimately reducing overall surgical procedure time [4]. In the same scope, Schulman et al. trained robots to tie knots using human demonstration via trajectory transferring [5]. In the modern era of aging populations, emerging ML technologies that involve ambulatory monitoring of the elderly are also becoming increasingly relevant. The latter include ML techniques integrated into wearable sensors for the detection of falls, optimized to differentiate between accidents and the activities of daily living. Six DL techniques were trained and their performance was compared in one study; the K-Nearest Neighbor (K-NN) and the Least Squares Method (LSM) classifiers showed the best outcomes [6].

The concept of personalized medicine is gaining widespread applicability in virtually all sectors of medicine. Large-scale trials aimed at integrating clinical, genetic, immune, and metabolic data underpinning personalized health have shown promising results. Phenotype-expression Association eXplorer (PEAX) is an analysis technique that integrates visual phenotype modeling with statistical data testing. It is an open-source tool that merges statistical analysis with interactive decision tree algorithms, allowing physicians to put together clinical and molecular information to improve prognosis and treatment response predictions. PEAX was used to investigate evidence of correlation between the presence of single nucleotide polymorphisms in the β-adrenergic receptor gene and treatment response in left-sided heart failure cases [7]. In another study, automated phenotyping of patients using DL on data in electronic health records showed improved accuracy for predicting mortality, length of hospital stay, and discharge [8].

Whole-slide imaging (WSI) has recently been cleared by the United States Food and Drug Administration (FDA) for primary clinical diagnostic evaluation [9]. The clearance of the Philips IntelliSite Pathology Solution marked a major milestone in DP and is expected to push the boundaries of computational innovation in healthcare. Recently, the FDA has given clearance for Leica Biosystems to begin marketing of the Aperio AT2 DX System for clinical diagnosis in the U.S [10]. These regulatory developments have ushered a steady increase in the recognition of the potential of DP to enhance pathologists’ productivity and accuracy. The details of such enhancements remain largely speculative, but they include facilitating access to subspecialty expertise, as well as integration of AI-based applications that can improve diagnosis, classification, tumor grading, and stain interpretation [11]. For instance, ConvNet achieved outstanding results in differentiating between benign and malignant skin lesions [12]. Similarly, Arvaniti et al. used a DL-based algorithm to automate and standardize Gleason grading of prostatic adenocarcinoma and showed promising results particularly in cases with heterogeneous Gleason patterns [13]. To the best of our knowledge, the largest survey that summarizes deep learning models in DP is by BenTaieb and Hamarneh [14]. It encompasses more than 84 studies, including nine cohorts. The analysis concluded that standardization of image acquisition is needed for breakthrough of DP deep learning models into clinical practice and workflow.

Interestingly, a review of the literature shows limited numbers of studies of DP applications in hematopathology. Some cited reasons include the need for high-power magnification and perceived reliance on oil immersion objectives [15]. Notwithstanding, the advent of new ML algorithms has increased interest in and applications of WSI and DP in hematopathology. In this review, we focus on the applications of these emerging technologies in diagnostic hematology and the evaluation of lymphoproliferative diseases.

## 2. Digital Microscopy for the Identification of Normal and Abnormal Peripheral Blood Elements

Accurate identification of peripheral blood elements and various leukocyte subsets are an essential part of the investigation of benign and malignant hematologic diseases. Manual analysis of peripheral blood smears is time-consuming, labor-intensive, and subjective. Several analyzers have been introduced into the market aimed at overcoming these limitations. In 2001, DiffMaster Octavia^TM^ received FDA approval to market a device capable of recognizing peripheral blood elements using image analysis. CellaVision DM96 automatic hematology analyzer was introduced in 2004, followed by the CellaVision DM1200 in 2009 [16]. The latest version of CellaVision DM9600 was first launched in 2014 [17]. Of note, Merino et al. recently reported a review summarizing the essential cytologic features for optimization of digital images analysis of blood cells, including geometric parameters, color, and texture characteristics [18].

Software improvements have permitted important advances in the detection of circulating poikilocytes using digital microscopy. The CellaVision Software groups RBCs into 21 morphological categories. The most consequential of these is the schistocyte group as identified on peripheral blood smears (PBS). As the detection of schistocytes raises concern of microangiopathic hemolytic anemia, particularly thrombotic thrombocytopenia purpura (TTP), it constitutes a potential critical finding that requires emergent medical attention. For this reason, sensitive and specific recognition of schistocytes on PBS using automated morphology-based analyzers has received particular attention. In studies comparing schistocyte counts between the CellaVision DM96 and conventional manual microscopy, the former showed high sensitivity but poor specificity requiring reclassification by an expert laboratory professional [19,20]. The low specificity can be attributed primarily to the broad range of morphologic forms that can fall within the schistocyte group, which lacks a well-defined sine qua non shape. In the latter situation, AI-based algorithms with a dynamic range for learning to recognize schistocytes hold promise. The CellaVision analyzer showed better performance in recognizing RBCs from patients with hereditary hemolytic anemia. In such a context, the presence of certain poikilocyte groups above particular thresholds were shown to be disease specific. For instance, the percentage of microcytes showed high sensitivity and specificity for RBC membrane disorders at a cutoff of 5.7% [21]. Teardrop cell identification by digital microscopy was also investigated with good outcomes [22].

**Figure 2:**
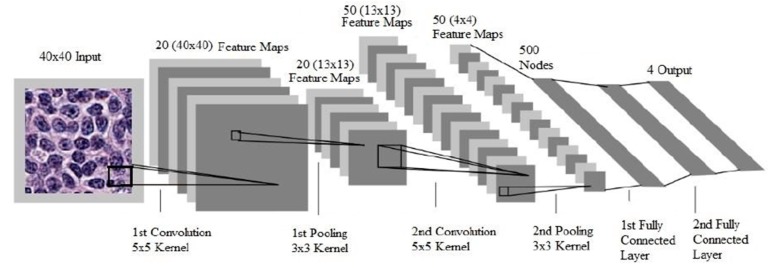
Processing pipeline of a convolutional neural network for the detection of visual categories in images. Example of a CNN model proposed previously by other authors. The convolutional layers perform feature extraction consecutively from the image patch to higher-level features. The pooling layers reduce image size by subsampling. The last fully connected layers provide prediction based on the given features. Reproduced with permission from Hanadi El Achi [32].

Characterization of lymphoid cells in PBS is a critical step that often forms the basis for further investigation of hematologic malignancies. Alferez et al. designed a method to improve the automatic classification of normal and mature B-cell neoplasms, which included chronic lymphocytic leukemia (CLL) and hairy cell leukemia (HCL). Images were segmented using the Watershed Transformation image processing technique to include 44 extracted features. The method developed showed high precision in automated classification of CLL and HCL cells [23]. In 2015, the same group expanded their method and applied the Watershed Transformation to include 113 extracted features combined with color image segmentation. They tested specimens from healthy individuals and patients with CLL, HCL, and mantle cell lymphoma (MCL), and achieved an accuracy of 98.07%. The precision, sensitivity, and specificity values were 99.7%, 97.5%, and 98.6%, respectively [24]. To expand the range and the precision of recognizing the lymphoid cells in the PB by automated digital microscopy systems, the investigators used the support vector algorithm that incorporated color and texture in addition to geometrical cytologic features. The accuracy for the identification of three categories, including reactive lymphocytes, normal lymphocytes, and abnormal lymphoid cells, was 97.67%. Further characterization of the abnormal lymphocytes into HCL, MCL, FL, CLL, and prolymphocytes diminished the accuracy to 91.23% [25]. In another proof-of-concept, underscoring the efficiency of automated PBS image analyzers, investigators categorized a set of features using digital image analysis to distinguish between reactive lymphocytes, blasts, and a wider array of abnormal lymphocytes, including CLL, B-cell prolymphocytic leukemia (B-PLL), HCL, splenic marginal zone lymphoma (SMZL), MCL, FL, T-cell prolymphocytic leukemia (T-PLL), T-large granular lymphocytic leukemia (T-PLL), and Sézary Syndrome cells. They included the highest number of extracted features, encompassing 27 related to geometry and 2649 related to color and texture, and succeeded in defining multiple sets of features that were specific for the different entities [26]. Other studies were conducted to train PBS image analyzers to subclassify blasts into myeloblasts or lymphoblasts. A support vector ML algorithm was used to test features selection techniques followed by the evaluation of different sets of features to ultimately identify the most specific sets for each category of blasts. The true positive rate for the identification of reactive lymphoid cells, myeloblasts, and lymphoblasts were 85%, 82%, and 74%, respectively [27]. Identification of normal blood elements and white blood cells classification is a major part of the daily routine work of laboratory technicians. Automation of this task would ultimately save time for more challenging duties. A convolutional neural network algorithm, which is currently the state-of-the-art in computer vision, was designed and tested to execute the task. The advantage of CNN over other deep learning techniques is its ability to deal with the enormous amount of features presented to the software for training. To overcome this obstacle, CNN has two additional layers: convolution layers, which perform feature extraction consecutively from the image patch to higher-level features, and the pooling layer, which reduces the image size of the convolutional layer by subsampling, while preserving the important information. Finally the last fully connected layers provide prediction and outputs based on the given features (Figure 2) [28]. Compared to classic ML algorithms, CNN showed better results in terms of precision of identification of four types of WBCs, including eosinophils, neutrophils, lymphocytes, and monocytes. The precision was as high as 93% when the detection was limited to mononuclear cells versus polynuclear cells, and dropped to 88% when the four classes are considered [29]. However, to be suitable for real-time object detection, the ML software should be able to identify normal and abnormal leukocytes at a fast pace. In this scope, a group from China, Wang and coworkers, conducted a project for the recognition of the leukocytes in the peripheral blood, exploring the outcomes of two single-stage detection frameworks: Single Shot Multibox Detector (SSD), and You Only Look Once (YOLOv3) pipelines. Both pipelines use a convolutional approach in which the network is able to identify all cells within an image in a single pass through the convent, giving them the characteristic of rapid detection. The ultimate aim of the group was to combine good sensitivity and accuracy with faster computational speed. Eleven categories of white blood cells were included. SSD, with an input size of 300 × 300, slightly out-performs YOLOv3 in terms of mean average precision (mAP) for the detection of 11 types of WBCs; the mAP being 93.1% and 92.25%, respectively. Moreover, the precision of detection was better for the blasts and mature white blood cells compared to the immature types, the accuracies being 97%, almost 100%, and 87%, respectively. In terms of inference time, YOLOv3 achieved outstanding outcomes, as the inference time was 14 ms per image compared to 53 ms per image for the SSD pipeline [30]. 

The application of DP for identification of PB abnormalities has grown beyond the detecting, counting, and classifying of blood elements to also incorporating the detection of *Plasmodium* and *Babesia* organisms. CellaVision DM96 was used to compare the detection rates of these species with conventional microscopy scanning. The parasite detection rate was 81% on regular microscopy and as high as 100% for the identification of *Plasmodium malariae* and *Babesia* species when parasitemia was ≥2.5%, and as low as 63% when parasitemia was <0.1% [31].

## 3. Digital Pathology for the Diagnosis of Acute and Chronic Leukemia in Peripheral Blood and Bone Marrow

The classification of leukemic diseases is based on refined criteria that rely on immunophenotypic, flow cytometric, cytogenetic, and mutation analysis results, alongside histologic and cytologic features. Against this backdrop, efforts are underway to develop tools for maximizing and optimizing the extraction of accurate and detailed information from PBS and bone marrow (BM) smears as additional ancillary diagnostic tools at baseline and for disease monitoring.

The lack of strong data on applications of DP in hematologic diseases can be attributed to technical limitations of WSI in this area, particularly the unavailability of three-dimensional images. Researchers from Stanford University started a new pipeline with super-resolution DP images for the interpretation of BM smears. The program consists of constructing a super-resolution image from multiple images to create a three-dimensional digital picture. The algorithm allowed a significant improvement in image sharpness and resolution when applied to BM aspirate smears [33].

The evaluation of BM biopsies starts with a proper assessment of the cellularity. The evaluation of cellularity is still subjective, and reported based on the visual estimate in correlation with the patient’s age. Currently, efforts to standardize the measurement of the BM cellularity using machine learning are promising. The HALO imaging software, an image analysis software that uses algorithms to report multiplexed morphological data on a cell-by-cell basis in a histology section, was used to assess the cellularity of BM trephine biopsies. The correlation with the visual estimate performed by expert hematopathologists was merely 81% [34]. On the other hand, VisioPharm image analysis platform applications for the diagnosis of lymphoproliferative diseases are limited, however the software was tested for quantitation of lymphocytic aggregates in Sjögren biopsies; the Bayesian-based algorithm designed matched the pathologists’ scoring in 100% of the cases [35].

Along with the other criteria, one of the major critical factors for the classification of hematologic malignancies is the BM differential cell counts (DCC); however, this count is time-consuming and subject to major inter- and intra-observer bias. Therefore, standardization and automation of DCC would tremendously improve the accuracy of the count. Efforts have been initiated to apply ML techniques to identify and classify normal BM elements, and promising results are emerging [36]. Further, enumeration of blasts in the BM, and distinguishing the different categories of these cells, are also pivotal factors for disease diagnosis and classification of acute leukemia [37]. An increasing number of studies related to diagnosing leukemia were conducted after 2010 using ML algorithms; they included the four common types of leukemia: acute lymphocytic leukemia (ALL), chronic lymphocytic leukemia (CLL), acute myeloid, leukemia (AML), and chronic myeloid leukemia (CML). Interestingly most of the researchers used the supervised algorithms of DL. Recently, investigators have been applying unsupervised methods to minimize the need for manual segmentation and feature extraction, which are time consuming tasks for operators, particularly in hematology and cytology. This is in line with conducting studies to apply ML in the hematopathology field [38]. 

Concomitantly, models for automated interpretation of flow cytometry (FC) results for diagnosing hematologic malignancies were developed. The drawback of training models for FC automated interpretation is the need for large numbers of abnormal cases. The “Data Augmentation” methods, such as random cropping, image rotation, and image inversion, are not applicable in FC plots. In spite of these obstacles, outstanding outcomes were achieved in terms of accuracy of the different models. For instance, Biehl et al., Manninen et al., and Dundar et al. applied generalized matrix relevance learning vector quantization, a regularized logistic regression model, and non-parametric Bayesian algorithms of deep learning, respectively, for the detection of AML by flow cytometry. The range of area under the curve fluctuated between 98% and 100%. Interestingly, another group achieved 99.6% accuracy in FC diagnosis of CLL [39,40,41,42]. Significant outcomes also resulted from applying the support vector machine method to identify the immunophenotypic patterns of malignant myeloid cells of CML and the normal/reactive neutrophils [43]. These findings make ML algorithms quite valuable for the interpretation of flow cytometry results, in particular for the diagnosis of CLL.

Several studies have attempted to create AI models using PBS and/or bone marrow histologic findings. ALL diagnosis received marked attention with a high number of studies that attempted not only to diagnose ALL but also to subtype it on morphologic grounds. Only bone marrow specimens were selected to detect and classify ALL using supervised ML models by Rehman et al. and Reta et al.; these groups achieved an overall accuracy of 98% and 92%, respectively, for sub-classification into L1, L2, and L3 vs. normal marrow [44,45]. Shafik et al. applied unsupervised models of Deep convolutional neural network to peripheral blood smears and demonstrated a sensitivity of 100% and a specificity of 98% for the detection of ALL, and a sensitivity of 97% and specificity of 99% for ALL sub-classification into L1, L2, L3 and distinction from normal [46]. Other groups trained supervised models for segmentation and classification of ALL, mainly the support vector machine (SVM) algorithm. The overall accuracy for the detection of lymphoblasts and differentiating them from reactive lymphoid cells ranged from 74% to 99%, with a sensitivity as high as 100% and a specificity up to 95% [33]. The best outcomes were obtained by Bhattacharjee et al., who collected 120 cases and used pattern recognition-based segmentation to train and compare the results of multiple classifiers, including artificial neural network (ANN), k-nearest neighbor (kNN), k-means, and support vector machine (SVM) [47]. The combination of both BM and PBS analysis for the automated diagnosis of leukemia achieved results comparable to PBS alone; however, further sub-classification showed sensitivity, specificity, and accuracy >90% for distinguishing L1, L2, and L3 ALL subtypes from non-neoplastic cells. A study of AI in ALL has also encompassed optimization of therapy using the Phenotypic Personalized Medicine Digital Health Platform, which identifies patient-specific factors that correlate drug dosage with phenotypic outputs. The algorithm demonstrated that adjusted dosing of combination chemotherapy could enhance treatment outcomes and maintenance therapy while reducing the amount of chemotherapy administered and ultimately the risk of side effects [48].

Automated detection and sub-categorization of acute myeloid leukemia (AML) was also investigated. Supervised algorithms and particularly pattern recognition-based segmentation methods were widely used along with SVM classifiers. The accuracy of algorithms used has ranged from 82% to 97% for the detection of myeloblasts. Reta et al. developed an algorithm to distinguish between the FAB subtypes of AML, and they reported an accuracy of 100% for diagnosing M2, M3, and M5 [40]. However, the new WHO classification of AML includes numerous entities based on genetic alterations as well as the precise histologic findings and immunophenotypic aberrations; hence, we believe that the main benefit of these preliminary studies is to identify the most efficient segmentation methods and classifier. Ultimately, gathering enough experience will make the digitalization process amenable to adding value to routine diagnostic evaluation and/or disease monitoring. 

Few reports investigated automated diagnosis of CLL or atypical CLL (aCLL) by morphology. A case report was published in 2014 by a team from the Memorial Sloan Kettering Cancer Center described a patient who presented with mild leukocytosis and lymphocytosis; the WBC differential by digital microscopy (Cellavision) revealed 60% lymphocytes and 26% large abnormal lymphoid cells, overall findings consistent with atypical CLL (aCLL) [49]. The report was presented as a proof-of-concept that digital microscopy is a fast screening tool to improve the identification of aCLL cases; particularly that this entity has a more aggressive prognosis. This method was further explored by another group that included a larger number of cases; however, the study highlighted a few pitfalls to be considered in automated diagnosis of aCLL. In particular, there was a higher risk to misclassify the atypical cells as plasma cells, monocytes, myelocytes, or blasts, leading to a risk of missing the diagnosis [50]. In 2015 and 2016, Alferez et al. developed two platforms for the automated recognition of atypical lymphoid cells, including CLL cells. They first used color features and the watershed transformation as criteria for segmentation and linear discriminant analysis for the recognition, using 1500 images for training. The accuracy in this study was 80% and the specificity was 98.6% for the identification of five types of lymphoid cells, which included normal lymphocytes, HCL, CLL, MCL, and B-PLL [23]. In 2016, the same group used 4000 images to detect the same entities as well as follicular lymphoma cells. They achieved an overall accuracy of 98% for the screening of normal lymphocytes, abnormal lymphoid cells, and reactive lymphocytes, and they demonstrated an accuracy of 91.23% for the classification of the abnormal lymphoid cells into specific disease entities (Table 1) [24].

## 4. Digital Pathology for the Diagnosis and Grading of Lymphoma:

The use of digital pathology in the diagnosis of lymphoid neoplasms in tissue has also been studied (Table 2). Recent projects have shown promising results using ML to detect lymphomas with WSI. Compared to other lymphoma entities, pathology diagnosis of follicular lymphoma (FL) was the subset with the highest number of studies. The very first projects aimed at digitalizing the grading of this entity. Currently the standardized risk stratification method is the histological grading system suggested by the World Health Organization (WHO). This method requires performing a count of the centroblasts per microscopic high power field (HPF). The average count helps classify the disease into one of the three grades: Grade I (0–5 CB/HPF) and Grade II (6–15 CB/HPF), which belong to the low-risk subset, and Grade III (>15 CB/HPF), a high-risk category [31]. However, the narrow ranges of the centroblast counts for the grading along with the subjectivity of the count can affect the grade of the disease, and consequently the prognosis and treatment. Inter-observer variation can reach up to a 41% lack of consensus between expert pathologists [43]. Since centroblast counts should be performed in neoplastic follicles, the generation of an automated system that accurately identifies the targeted follicles is a fundamental step toward standardization. It was reported that digital reading from WSI with preselected regions improved inter-reader agreement, with only 5.9% lacking consensus for centroblast enumeration [51]. However, fields were randomly selected by one of the pathologists, and hence might also be affected by subjectivity bias through including the zones that look histologically remarkable or of higher grade.

Other projects used immunohistochemistry (IHC) slides for particular markers to identify the neoplastic follicles in a FL case and perform an appropriate selection of the high-power fields for an accurate count of the centroblasts. Samsi et al. focused on developing a method to detect neoplastic follicles based on IHC for B-cell markers in a lymph node, including CD10 and CD20. Comparison of automated segmentation of the follicles with manual segmentation showed an accuracy of 87%. Identification of all the follicles in a tissue section can help perform grading on a thorough examination of the available tissue instead of limiting the centroblasts count to only 10 HPF, ultimately improving the accuracy of grading FL [52,53]. Oger et al. also created a system using CD20 to delineate the follicles and refine the results by mapping follicle boundaries on high-resolution H&E images [54]. Few projects focused on using only the H&E images solely to identify the follicles. Belkacem-Boussaid refined the automated segmentation of the follicles by using the concavity index and a recursive watershed operation to reduce the over-segmentation bias. The accuracy of their method was 78% [55]. The same group has also published an automated method to identify centroblast (CB) cells independently of the digital identification of the follicles. The method used both geometric and texture features extraction; the accuracy of distinguishing centroblast from non-centroblasts was 82% [56]. Overall, the precision of the different preliminary methods designated for the automation of the FL grading is still far from being implemented for real-life cases; more accurate platforms and algorithms need to be developed. The most recent project conducted by the Ohio University team for the classification of the FL was published in 2015. They described a Follicular Lymphoma Grading System (FLAGS) that automatically identifies multiple (>10) candidate fields in the tissue section that are suitable for grading; the method uses H&E and CD20 stains in combination. The identification step is followed by a classification of the selected fields into a high or low grade based on the number of the centroblasts detected. The accuracy of the method was 80% [57]. It should be pointed out that for simplification purposes all the authors selected straightforward cases of FL without including the diffuse pattern, therefore these methods will not classify and grade all the cases with the same high accuracy and precision.

The earliest efforts to standardize the FL grading were started since 2009 by Sertel et al. who used a different perspective; they developed a combined approach using cytologic components and the spatial distribution of the areas of interest with color texture analysis. They observed that a better classification of the low-grade entities relies on particular color features; hence, the method was very successful in identifying the Grade III subsets with a 98.9% sensitivity and 98.7% specificity, but more standardization was needed for the lower grade FL. These findings highlight a pitfall of the DP related to the H&E staining, as well as the quality of the WSI, conferring a subjective aspect to the automated diagnosis [58]. To overcome this limitation, the same group of researchers developed another system for the detection of centroblasts; the method uses *a unitone conversion* to obtain a single channel image that has the highest contrast. Moreover, to refine their results, they used a two-step procedure: in the very first step, they identified the non-centroblasts, followed by the detection of the centroblasts. The detection accuracy of the method was 80.7% [59]. Overall, the efforts of optimizing the automation of hematologic malignancies grading serve, at least in the current state, as potential methods for tele-consultation between different institutions for second opinions and better patient care. 

Algorithms based on IHC staining have been established to guide the subclassification of diffuse large B-cell lymphoma (DLBC) on the basis of the cell-of-origin. However, it did not show a high concordance with the gene expression profiles [60,61]. Da Costa developed a new algorithm for DLBCL IHC classification through a machine learning method J48, to include CD10, MUM1, FOXP1, and BCL-6 into an IHC automated classification algorithm. Interestingly, 91.6% of the cases were correctly classified as GC or non-GC, showing a high concordance with the GEP and the prognostic significance [62]. A group from Mexico recently published an ML approach based on a combination of IHC antibodies included in the different DLBCL algorithms. They performed a comparison between the new algorithm and the previously available ones; they used multiple ML structures, including artificial neural networks and support vector machine to identify the best classifier. Their algorithm showed a 94% accuracy, 93% specificity, and 95% sensitivity, highlighting a high agreement with GEP [63]. Another interesting study was conducted in China, aiming to investigate the concordance of the IHC molecular subtype among six known IHC algorithms, and to evaluate the clinical significance of the different algorithms in patients treated with CHOP/R-CHOP (rituximab, cyclophosphamide, doxorubicin, vincristine, and prednisone) chemotherapy regimens. They compared the outcomes of 381 cases of de novo DLBCL, not otherwise specified (NOS) patients, to the GEP results. The support vector machine algorithm was used to compare the results of the different algorithms. The study showed high concordance rates with the GEP for the Choi and Visco–Young algorithms, which suggests the ability of these protocols to consistently separate DLBCL–NOS into GCB and non-GCB subtypes. More studies on the same track can help identify the most accurate method that adequately correlates with the prognostic effect of the DLBCL subtypes [64]. These studies are a proof-of-concept of tremendous importance of AI to make the classification of the DLBCL subsets easier and more precise and for exploring the efficacy of the different chemotherapy regimens for each sub-category. 

Currently, the clinical risk stratification of DLBCL is based on the International Prognostic Index (IPI), the revised IPI, and the National Comprehensive Cancer Network IPI (NCCN-IPI). These models are based on clinical and laboratory data. Interestingly, a European group recently included more variables and clinical information to the previous algorithms to develop a new model for the prediction of the prognosis of DLBCL. The new model is based on ML techniques and is available online (https://lymphomapredictor.org) [65]. Finally, treatment resistance to R-CHOP has also been investigated by the dint of AI methods. 2D and 3D CT radiographic analysis with ML techniques based on random forests (RF) and SVM were tested for constructing the prediction models. Lymph nodes sections from a patient with known treatment resistance were contoured and segmented before getting analyzed by the platforms. The models provided high prediction accuracy for treatment resistance [66]. Hence, AI is finding its way to fulfill the need of identifying DLBCL patients that might have treatment resistance to ultimately avoid toxicity from ineffective drugs. 

With regard to the application of AI for the diagnosis of lymphoma, available studies remain limited. To our knowledge, only a single study has explored how DL can be used to accurately classify cases into one of four categories: benign lymph node, DLBCL, BL, and SLL. The convolutional neural network algorithm was used to build the lymphoma diagnostic model for these categories based on H&E images. The method’s outcomes showed an excellent diagnostic accuracy of 100% [32]. 

The advent of immunotherapy and targeted therapy urges pathologists and oncologists to understand the different pathways of diseases, and the interaction of the cellular markers. This can be achieved via studying the interaction of the multiple biomarkers expressed by tumor cells. Few hematologic entities are diagnosed based on the pattern of co-expression of different membranous, cytoplasmic, or intra-nuclear markers. In the same scope, pinpointing small micro-metastasis in lymph nodes can be challenging; immunostaining of the different components of the lymph node simultaneously would highlight any abnormality undetectable on H&E sections. Therefore, multiplexed immunofluorescence for the study of tumor tissues will find a solid base for advancement in lymphoproliferative diseases. A review article published recently cited the different techniques for multiplexing. The available staining platforms encompass Multiplex staining bleaching techniques, Multiplex signal amplification techniques, and mass spectrometry imaging. Of these the MultiOmyx platform, which belongs to the staining techniques, allows the analysis of up to 60 biomarkers in a single slide; the same apply to the Multiplexed ion beam imaging, which can analyze up to 100 biomarkers simultaneously; however, the staining time is longer for both methods. Numerous automated scanning products are available in the market, such as AxioVision MosaiX, Multiplexed ion beam imaging, and MALDI–TOF mass spectrometry. Clear and neat staining and scanning techniques are of utmost importance for a better analysis of the tissue, particularly when the available specimen is limited to a small core biopsy [67].

## 5. Conclusions and Future Directions

Multiple studies have applied ML tools to diagnose hematologic diseases. Some have achieved high diagnostic accuracy. However, our review indicates that studies in this field remain limited. The outcomes of any digitalization system will ultimately require review/supervision by a pathologist who will approve or disaffirm the machine-derived results, taking into consideration histologic findings, clinical presentation, and other contingent factors. As such, the primary aim of using DP should be to facilitate and standardize the diagnostic process to complement and aid human activities in this space.

## Figures and Tables

**Table 1:** Studies utilizing machine learning (ML) for hematologic malignancies. PB, peripheral blood; LDA, linear discriminant analysis; HC, hairy cells; MC, mantle cells; FL, follicular lymphoma; CLL, chronic lymphocytic leukemia; SLA, supervised learning algorithms; SVM, support vector machine; BM, bone marrow; ALL, acute lymphocytic leukemia; AML, acute myelocytic leukemia; CML, chronic myeloid leukemia; KNN, K-nearest neighbor; RF, random forest; SL, simple logistic; RC, random committee; CNN, convolutional neural network; DCNN, deep convolutional neural network; ANN, artificial neural network; VE, visual estimates; DIA, digital image analysis; GMLVQ, generalized matrix relevance learning vector quantization; LDA, linear discriminant analysis; BC, Bayesian clustering; ASPIRE, anomalous sample phenotype identification with random effects; FC, flow cytometry; AUC, area under the curve; ICC, Intra-class correlation.

Study (Reference)	Entity of Interest	Tissue	Objective	Segmentation Identification Method	Classifier	Number of Images	Accuracy
Alferez et al. [25]	Lymphoid cells	PB	Recognition of atypical lymphoid cells	Clustering of color components—watershed transformation—pattern recognition-based	LDA	4389	98% accuracy 97.5% sensitivity 98.6% specificity
Alferez et al. [24]	Lymphoid cells	PB	Normal, reactive, HC, MC, FL, CLL, prolymphocytes	Geometry, new color and texture features—pattern recognition-based	SVM	1500	91%
Salah [38] metanalysis	ALL, AML, CLL, CML		Diagnosis of leukemia		22 studies SLA		
Ni et al. [43]	CML	LN	Identify malignant myeloid cells of CML		SVM	9 cases	≤95.80% specificity ≤95.30% sensitivity
Reta.et al. [45]	ALL-AML	BM	Distinction ALL vs AML and sub-classification of ALL	Pattern recognition-based	KNN, RF, SL, SVM, RC	633	94% accuracy (overall) 92% AML vs. ALL
Rehman et al. [44]	ALL	BM	Sub-classification of ALL	Threshold-based method	CNN	330	97.78%
Shafique et al. [46]	ALL	PB	Detection and classification of ALL	NA	DCNN	760 after augmentation	99% accuracy for detection 96% for classification
Bhattacharjee et al. [47]	ALL	PB	Detection of ALL	Pattern recognition-based	ANN, KNN, k-means, SVM		100% sensitivity 95% specificity
Hagiya et al. [34]	BM cellularity	BM	Assess relatedness between VE and DIA		Aperio AT2 Scanscope	165 cases	0.81 ICC
Biehl et al. [39]	FC for AML	PB, BM	Classification of AML	NA	GMLVQ	179 cases	100%
Manninen et al. [40]	FC for AML	PB, BM	Classification of AML	NA	LDA	359 cases	100%
Dundar et al. [41]	FC for AML	PB	Classification of AML	N/A	ASPIRE	50,000 using the resampling technique	99% AUC
Lakoumentas [42]	FC for CLL	PB	FC diagnosis of CLL	NA	BC		99%

**Table 2:** Studies utilizing ML for mature lymphoproliferative diagnosis, grading, and prognostication. WSI, whole slide image; RW, recursive watershed; DLBCL, diffuse large B-cell lymphoma, LN, lymph node, FL, follicular lymphoma; H&E, hematoxylin and eosin; IHC, immunohistochemistry; CI, concavity index; FLAGS, Follicular Lymphoma Grading System; GMM, Gaussian mixture model; EM, expectation maximization; ANN, artificial neural networks; SVM, support vector machine; RF, random forests; CNN, convolutional neural network.

Study (Reference)	Entity of Interest	Tissue	Objective	Segmentation Identification Method	Classifier	Number of Images/Cases	Accuracy
Lozanski et al. [51]	FL (grading)	LN	Agreement between glass slide and WSI reading	N/A	N/A	17 cases	95%
Samsi et al. [52,53]	FL	LN	Detection of follicles by IHC (CD10/CD20)	Iterative watershed, color and texture features	Unsupervised K-means clustering algorithm	8 images/ 12 images	87%
Oger et al. [54]	FL	LN	Detection of follicles by IHC (CD20)	Comparison of manual and automated segmentation	k-means classifier	12	
Belkacem-Boussaid [55]	FL	LN	Detection of follicles by H&E	Region-based segmentation, CI calculation, RW	NA		78%
Belkacem-Boussaid [56]	FL	LN	Identification of centroblasts	Geometric and texture features extraction	Supervised quadratic discriminant analysis	436 images	82%
Fauzi [57]	FL	LN	Grading of FL (H&E, CD20)	Geometric and color features; Manual extraction	k-nearest neighbor classifier—FLAGS	20 slides	80%
Sertel et al. [58]	FL	LN	Grading of FL (H&E)	K-means clustering algorithm and spatial distribution	Bayesian classifier	510 images	98.9% sensitivity 98.7% specificity for grade III
Sertel et al. [59]	FL	LN		GMM and EM unitone conversion of colors		100 images	80%
Da Costa et al. [62]	DLBCL	LN	Sub-classification of DLBCL (GC/non-GC)	N/A	J48 (WEKA package)	475 cases	92%
Perfecto-Avalos et al. [63]	DLBCL	LN	Sub-classification of DLBCL (GC/non-GC)	N/A	ANN and SVM	49 patients	94% accuracy 93% specificity 95% sensitivity
Zhao et al. [64]	DLBCL	LN	Outcome of patients after treatment based on the molecular subtyping algorithms	N/A	SVM	855 cases	94%
Biccler et al. [65]	DLBCL	LN	Prediction of prognosis	N/A	Stacking approach of ML	5173 cases	Excellent concordance
Santiago et al. [66]	DLBCL	LN	Treatment resistance to R-CHOP	Manual contouring	2D and 3D CT radiomic analysis with RF and SVM	254 lymph nodes	75% accuracy 80% sensitivity 69% specificity Superior results with RS
El Achi et al. [32]	Lymphoma	LN	Diagnosis of four lymphoma subsets	Unsupervised	CNN	2560 images	100%
